# Anticancer Applications of Essential Oils Formulated into Lipid-Based Delivery Nanosystems

**DOI:** 10.3390/pharmaceutics14122681

**Published:** 2022-12-01

**Authors:** Josef Jampilek, Katarina Kralova

**Affiliations:** 1 Department of Analytical Chemistry, Faculty of Natural Sciences, Comenius University, Ilkovicova 6, 842 15 Bratislava, Slovakia; 2 Department of Chemical Biology, Faculty of Science, Palacky University Olomouc, Slechtitelu 27, 783 71 Olomouc, Czech Republic; 3 Institute of Chemistry, Faculty of Natural Sciences, Comenius University, Ilkovicova 6, 842 15 Bratislava, Slovakia

**Keywords:** nanoemulsions, liposomes, solid lipid nanoparticles, nanostructured lipid carriers, essential oils, herbal drugs, anticancer activity

## Abstract

The use of natural compounds is becoming increasingly popular among patients, and there is a renewed interest among scientists in nature-based bioactive agents. Traditionally, herbal drugs can be taken directly in the form of teas/decoctions/infusions or as standardized extracts. However, the disadvantages of natural compounds, especially essential oils, are their instability, limited bioavailability, volatility, and often irritant/allergenic potential. However, these active substances can be stabilized by encapsulation and administered in the form of nanoparticles. This brief overview summarizes the latest results of the application of nanoemulsions, liposomes, solid lipid nanoparticles, and nanostructured lipid carriers used as drug delivery systems of herbal essential oils or used directly for their individual secondary metabolites applicable in cancer therapy. Although the discussed bioactive agents are not typical compounds used as anticancer agents, after inclusion into the aforesaid formulations improving their stability and bioavailability and/or therapeutic profile, they indicated anti-tumor activity and became interesting agents with cancer treatment potential. In addition, co-encapsulation of essential oils with synthetic anticancer drugs into nanoformulations with the aim to achieve synergistic effect in chemotherapy is discussed.

## 1. Introduction

Drug design and discovery are a very complicated process [1,2,3,4,5] with an uncertain result of a successful drug launch in the market [1,6,7,8]. Beyond the pharmacological aspect, the pharmacokinetic behavior of the molecules must also be taken into account for successful development [1,2,7,9,10]. In addition to synthetic molecules, another way to “invent” new drugs is inspiration from nature, in particular, secondary metabolites of living organisms. Their mixtures or isolated components/entities can be tested, and initially natural substances can be subsequently modified by chemical [1,2,9,11,12,13,14,15,16,17,18,19,20,21] or technological (galenical) [9,22,23,24] methods. One of the important sources of secondary plant metabolites are essential oils (EOs), which play a key role in plant protection and occupy a prominent place in folk medicine worldwide with their diverse biological activities, including antibacterial, antiviral, antioxidant, and anticancer properties, which predestine them to be used alone or in combination with synthetic drugs against numerous diseases, including cancer, or in aromatherapy. Thus, EOs represent an important group of substances with a long history of traditional applications that are widely used in the food, pharmaceutical, agricultural, and cosmetic industries [25,26,27,28,29].

Recently, the application of nanotechnologies has become very popular in medicinal products used in therapy and diagnostics [22,30,31,32,33,34,35]. According to physical sizes, any particles < 1000 nm are considered nanoparticles (NPs) [30]; industrially, NPs are particles < 100 nm [36,37], while in drug technology, submicron-size particles, i.e., sized in the range of 100–500 nm (most often 100–200 nm), are considered NPs [38,39,40,41]. There are many types of nanosystems used in biomedicine based on the materials they are produced from. Generally, they can be divided into inorganic, organic, and mixed or into degradable and stable/non-metabolizable in biological systems. There are a great many described preparation methods. Historically, nanoparticles were actually colloids prepared by precipitation. At the beginning of the new millennium, NPs could also be prepared by nanomilling in special nanomills in pilot and operation terms [42,43,44,45,46,47,48,49,50,51,52,53,54,55,56]. At present, lipid-based NPs are prepared by, e.g., homogenization, extrusion, cross flow injection, or microfluidic technologies [22,30,46,47,57,58,59,60,61,62,63,64,65].

The incorporation of bioactive compounds into nanosized matrices has a positive influence on their chemical stability. Moreover, surface modification allows one to engineer nanosystems with a defined time of circulation in the circulatory system or specific (passive, active) uptake in certain organs or only in damaged tissues. Thus, systemic drug toxicity is reduced. Lastly, drug-stabilizing matrices themselves can potentiate drug activity and modify it, thereby increasing its efficacy or overcoming cell resistance mechanisms [38,41,44,46,47,54,55,56,66,67,68,69,70,71]. These additional benefits of nanoformulations are used especially in formulations of anti-infective and anticancer drugs, and technologically, new generations of old molecules with improved therapeutic and safety profiles are entering the market [40,71,72,73,74].

The authors consider it needless to discuss further general issues/knowledge related to nanoparticles/nanoformulations (such as preparation, targeting, mechanisms of permeations into cells, etc.), because a lot of papers and books were written all over in this regard (see Refs. [30,31,32,33,34,35,38,39,40,41,43,44,45,46,47,48,49,50,51,52,53,54,55,56,57,58,59,60,61,62,63,64,65,67,68,69,70,71,75]); therefore, all respective information can be found in any available literature and is outside the scope of this review. By contrast, this brief overview summarizes the latest results of the application of nanoemulsions (NEs), liposomes, solid lipid nanoparticles (SLNPs), and nanostructured lipid carriers (NLCs) used as drug delivery systems of herbal essential oils (EOs) or directly for their individual secondary metabolites. Although the discussed bioactive agents are not typical compounds used as anticancer agents, after inclusion into the aforesaid formulations improving their stability and bioavailability and/or therapeutic profile, they demonstrated anti-tumor activity and became interesting agents with cancer treatment potential.

## 2. Herbal Medicinal Compounds

**Figure 1:**
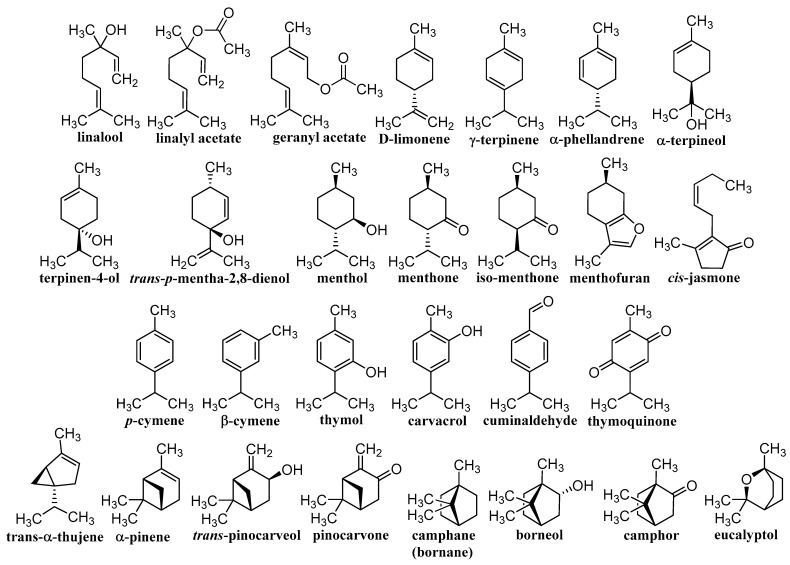
Structures of individual discussed monoterpenes.

**Figure 2:**
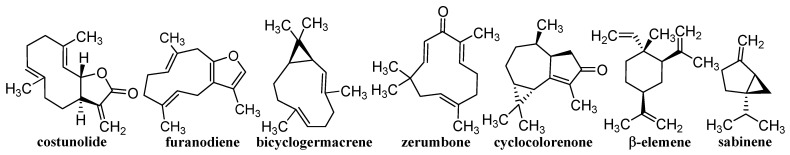
Structures of individual discussed sesquiterpenes.

Herbal drugs or medicinal plants have been used in traditional folk medicine for thousands of years [11,12,13]. Therapeutically, certain parts of plants are used, or they are extracted and complex extracts are used, or individual components are subsequently isolated. Effective compounds arise as a result of specific metabolism and belong to the so-called secondary metabolites, of which saccharides and their derivatives glycosides, EOs, steroids, lipids, bitters, alkaloids, tannins, flavonoids, pigments, compounds with hormonal action, proteins, peptides, and vitamins are used. This is a wide range of structurally variable molecules with various biogeneses, mechanisms of action, and activities [20,76,77,78]. The contribution discusses different terpenoids shown in Figure 1 and Figure 2, whose unifying element is their anticancer activity confirmed on a nanoscale. Classical nature-based anticancer drugs, which or whose semi-synthetic modifications have been used in cancer treatment for a long time—e.g., taxanes, vinca alkaloids, camptothecin-based derivatives, podophyllotoxin, colchicine, and anthracyclines—will not be presented.

EOs contain terpenoids, which are small fat-soluble organic molecules that can be absorbed through the skin or nasal mucosa into the systemic circulation and cross the blood-brain barrier. Therefore, topical application or inhalation of EO can also produce a systemic effect. They are volatile and not sufficiently stable in light and ambient temperature. They can be used directly with the whole plant or extracted from plants/plant parts, and then these concentrated extracts are used for healing purposes. EOs occupy a prominent place in traditional and folk medicine around the world. Due to a high content of volatile aromatic compounds, they smell great, are used in aromatherapy to reduce stress and anxiety, have powerful antimicrobial properties, help induce sleep and improve sleep quality, improve cognitive function, and have the ability to lower blood sugar. EOs are also powerful antioxidants that help prevent free radical damage to cells, hence they can contribute to cancer prevention [79,80]. Their cytotoxicity preventing tumor growth is manifested by a wide spectrum of mechanisms of action. Essential oils have been shown to have cancer cell-targeting activity and are able to enhance the effectiveness of commonly used chemotherapy drugs while demonstrating pro-immune functions when administered to a cancer patient [81,82].

**Figure 3:**
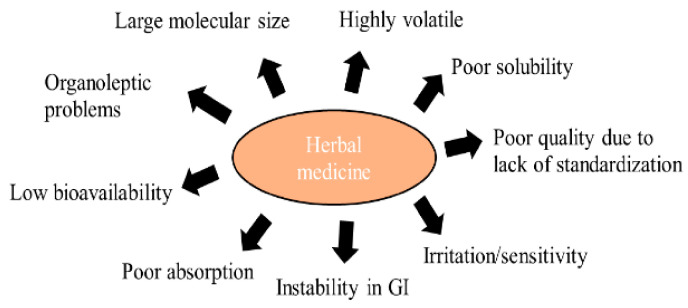
Limitation of herbal medicines. Adapted from [85].

Although the use of nature-based drugs has many disadvantages, society considers it more favorably than the consumption/application of synthetic drugs. There are certainly differences between the use of a dried plant drug in the form of tea/decoction/infusion, a standardized extract, EO, and isolated secondary metabolites. The disadvantages of nature-based drugs are illustrated in Figure 3. Nevertheless, a standardized plant extract/EO or an isolated secondary metabolite can be formulated to various sophisticated drug formulations, similarly as synthetic drugs, or can be co-formulated with them. Natural molecules that are adjusted technologically in such a way to achieve increased stability, bioavailability, and improved activity can be successfully applied in anticancer indications [83,84,85,86].

The advantages of microemulsions, NEs, liposomes, microparticles, SLNPs, and self nanoemulsified drug delivery systems as delivery systems of herbal materials were overviewed by Severino et al. [84] and Baena-Aristizabal [87]. The use of liposomes, NEs, ethosomes, phytosomes, and lipid NPs as delivery systems of encapsulated plant natural extracts or their secondary metabolites with pharmaceutical activity, which exhibit a sustained release, improved stability, and ameliorated therapeutic effect and can ensure protection from toxicity, was discussed by Verma et al. [88] and Mahomoodally et al. [89].

## 3. Nanoemulsions of Essential Oils

**Figure 4:**
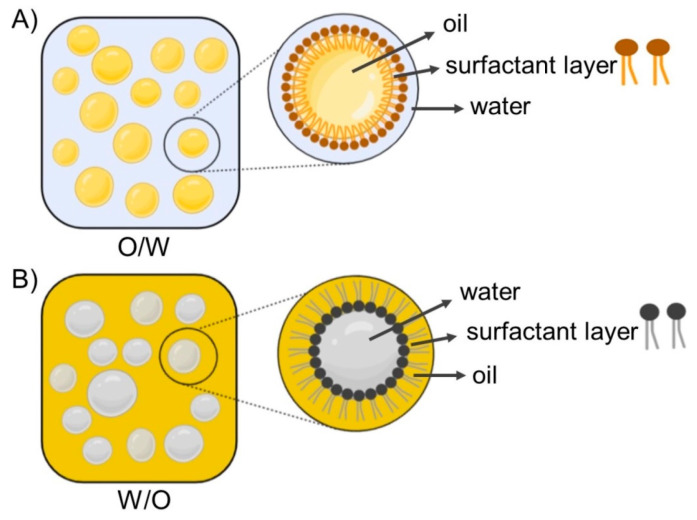
Schematic illustrations of oil-in-water (O/W) (**A**) and water-in-oil (W/O) (**B**) emulsions, representing micelle structure dispersed in continuous phase for each system. Adapted from [95].

Nanoemulsion (NE) is defined as a heterogeneous system consisting of two immiscible liquids dispersed in one another, where the emulsion particle size is less than 1000 nm [90]; however, the average particle size is usually within 100–500 nm. In practice, the particle size of NEs ranges from 20 to 200 nm and is characterized by a narrow particle size distribution [91,92]. In principle, two types of nanoemulsions are distinguished: the type of oil dispersed in water (o/w) and the inverse type (w/o) [93]. NEs are often referred to as translucent or transparent, while classic emulsions are characterized as non-transparent or milky [94]. Schematic illustrations of emulsions are shown in Figure 4 [95]. Due to the attractive properties of NEs, such as small sizes, high surface area, improved dispersion of hydrophobic active ingredients, enhanced absorption, and ability for site-specific or targeted delivery, NEs have become widely used as delivery systems for drugs and dietary supplements, and also in cosmetics [49,50,51,53,54,55,56,75,94,96,97,98,99]. High- and low-energy methods are used to prepare NEs, including high-pressure homogenization, ultrasound, phase inversion temperature, and emulsion inversion point [51,54,55,56,75,84]. Contributions describing in detail the physical characteristics, composition, and methods of preparations of NEs were published recently [94,100].

Recent progress in the use of NEs as delivery systems contributing to the improved efficacy of EOs and antimicrobials used in the treatment of infectious diseases via antimicrobial therapy was presented by Garcia et al. [101]. The advantage of herbal nanoformulations over conventional anticancer drugs is their lower toxicity, as well as their improved bioavailability and therapeutic efficacy [102]. Recent findings related to NEs as delivery systems for bioactive compounds, originating from fruit and vegetable waste and showing numerous biological properties, including anticancer activity, were summarized by Saini et al. [103].

### 3.1. Isolated Components of Essential Oils

A NE of carvacrol, a monoterpenoid phenol occurring in *Thymus* spp., with mean droplet size 105.5–169.8 nm caused reactive oxygen species (ROS) production in adenocarcinoma human alveolar basal epithelial cells A549, resulting in the activation of crucial apoptosis regulators (p-JNK, BAX, and BCL2), release of cytochrome c, and activation of the caspase cascade, whereby mitochondrial ROS were involved in the cell death; the powerful antitumor activity of the NEs was also observed in vivo using an athymic nude mice model [104]. Another carvacrol NE prepared using Tween 80 with average particle size 14–30 nm was reported to exhibit cytoprotective effect against cisplatin (CDDP)-induced nephrotoxicity in albino rats [105]. Another carvacrol NE with mean particle size 99.1 nm and zeta potential −29.89 mV induced an increased expression of apoptotic proteins in doxorubicin (DOX)-resistant A549 lung carcinoma cells, resulting in apoptosis, and caused cell cycle arrest via reducing the expression of CDK2, CDK4, CDK6, cyclin E, and cyclin D1 proteins and enhancing the expression of p21 protein; it also inhibited autophagy via down-regulating autophagy markers ATG5 and ATG7 and upregulating p62 [106]. A review article discussing nanocarriers used as a delivery system for d-limonene, one of the main bioactive ingredients in citrus peels, showing anticancer activity, was published by Akhavan-Mahdavi et al. [107]. A NE stabilized by in situ self-assembled natural oil/native cyclodextrin complexes encapsulating costunolide (CTD), a metabolite extracted from plant species *Saussurea*, *Aucklandia*, and *Inula* showing excellent anticancer activity with mean particle size 199.56 nm was found to exhibit considerably higher anticancer activity against A549 lung cells than non-encapsulated CTD, causing pronounced cell cycle arrest at the S phase; a remarkably higher expression of caspase-3, BAX, and BCL-2, and p53 mRNA expression; and reduced activity of tumor necrosis factor alpha (TNF-α) and nuclear factor-kappa B (NF-κB) compared to free CTD [108].

By loading bleomycin, an antibiotic, in a cinnamon oil NE with average particle size 119.60 ± 1.20 nm and zeta potential −0.913 ± 0.001 mV, the resulting NE particle size increased to 524.33 ± 1.10 nm and zeta potential achieved 0.537 ± 0.002 mV; such NE showed a higher apoptotic effect on HeLa cervical cancer cells than free bleomycin [109]. Similarly, an ifosfamide-loaded camphor EO NE (96.235 ± 9.00 nm; zeta potential −22.00 ± 0.49 mV) showed a higher cytotoxic effect on MCF-7 breast cancer cells and HeLa cervical cancer cells than a camphor EO NE (34.975 ± 9.35 nm; zeta potential −13.75 ± 1.06 mV) and free ifosfamide [110].

### 3.2. Essential Oils and Plant Extracts

A NE of *Carum carvi* EO exhibited apoptotic and cytotoxic effects on colon cancer cells (HT-29); it reduced more effectively the viability of HT-29 cancer cells (IC_50_: 12.5 μg/mL) compared to HUVEC normal cells (50 μg/mL), considerably upregulated caspase-3 gene expression, and did not show undesirable side effects. The strong apoptotic activity of *Carum carvi* EO NE predestines it to be applied as food supplement also [111]. A NE of *Cuminum cyminum* seed EO with average droplet size 10.4 ± 0.5 nm reduced the viability of a tongue carcinoma cell line (SAS; IC_50_: 1.5 μL/mL), causing early induction of apoptosis, and diminished colony formation; the NE also showed superb antibacterial activity [112]. NEs fabricated using *Cuminum cyminum* L. tinctures with mean particle size 24 nm and zeta potential −26.43 ± 9.87 mV showed a dose-dependent inhibition of angiogenesis via reducing the number and length of blood vessels, presumably by inhibiting the expression of vascular endothelial growth factor (VEGF) and VEGF receptor (VEGF-R) genes, along with superb antibacterial and antioxidant activity [113]. *Anethum graveolens* EO NEs were reported to reduce the viability of A549 cells and could be considered as an exclusive apoptotic inducer in these lung cancer cells [114]. A *Coriandrum sativum* EO nanoemulgel prepared using a self-nanoemulsifying technique showed better anticancer activity than the crude EO achieving IC_50_ values of 28.84 μg/mL, 28.18 μg/mL, and 24.54 μg/mL against breast cancer cells (MCF-7), hepatocellular carcinoma cells (Hep3B), and human cervical epithelioid carcinoma cells (HeLa), respectively [115]. A *Ferula assa-foetida* EO NE exhibited remarkable cytotoxic, apoptotic, and anti-angiogenic impacts on MCF-7 cancer cells and caused increasing destruction of the murine mammary glands’ cancer tissue, suggesting that it might be used as an effective agent to treat breast cancer [116]. A *Ferula gummosa* EO NE with spherical droplets of 24.6 nm and zeta potential of −28.5 mV synthesized at a concentration of 2.9 μg/mL exhibited approximately 50% inhibition of HT-29 cells, but did not inhibit normal cells up to a concentration of 4 μg/mL. The NE increased caspase-3, caspase-9, and BAX and decreased BCL-2 gene expression and induced apoptosis, inhibited angiogenesis and showed an additive effect on the expression of antioxidant genes. Moreover, the NE was able to reduce tumor volume by 69.72% in 14 days in the in vivo murine colon cancer model [117]. A *Heracleum persicum* EO NE inhibited the proliferation of MDA-MB-231 breast cancer cells (IC_50_: 2.32 μg/mL), and the application of 1.5 μL/mL considerably suppressed cell migration, while doses of 1.5, 2.5, and 3.5 μg/mL pronouncedly upregulated caspase-3, suggesting that the NE was able to induce apoptosis death in MDA-MB-231 cells and did not exhibit cytotoxic effects in the liver, kidney, and jejunum of mice. Mice fed with 10 and 20 mg NE/kg body weight were characterized with a pronouncedly upregulated expression of superoxide dismutase (SOD), catalase (CAT), and glutathione peroxidase (GPx) genes in the liver, but showed ameliorated villus height, villus width, crypt depth, and goblet cells [118]. A NE of *Apium graveolens* seed oil with droplet diameter 23.4 ± 1.80 nm showed cytotoxic effect against oral squamous cell carcinoma cells (IC_50_: 1.4 μL/mL) and pronouncedly reduced the proliferation of cancer cells via suppressing anchorage-independent cell growth, disrupting colony formation, and inducing apoptosis of cancer cells. Moreover, the NE also exhibited antibacterial activity against *Staphylococcus aureus*, causing lipid membrane fusion and cytoplasmic leakage, resulting in complete destruction of pathogen [119].

An *Origanum vulgare* EO NE showed anticancer effects against prostate cancer cell lines (PC3), reduced the density and shape of cells, and caused cell shrinkage. Moreover, it considerably diminished the accumulation of lipid droplets, fatty acid synthase, and sterol regulatory element-binding protein; remarkably upregulated BAX (B-cell lymphoma 2 (BCL2) associated X) and caspase-3 expression; and reduced the transcript level of BCL2 leading to apoptosis [120]. A NE of *Origanum glandulosum* Desf. EO fabricated by high-pressure homogenization (HPH) exhibited a lower cytotoxic effect on the liver cancer cell line HepG2 than the free EO, which was reflected in IC_50_ values of 131.6 μg/mL and 73.13 μg/mL, respectively, suggesting that HPH adversely affected the content of carvacrol, thymol, and other active compounds, thereby altering the content of volatile compounds [121]. A NE of *Mentha piperita* EO containing menthol (31.0%), menthone (22.1%), camphane (7.0%), menthofuran (6.0%), and iso-menthone (5.8%), with average particle size of 136 ± 2 nm as major constituents, exhibited anticancer effects against MCF-7, MDA-MB-231, and MDA-MB-468 breast cancer cells, and after 24-h exposure to the NE formulation, the observed effect was considerably higher than that after 72 h exposure to the free EO. Moreover, *M. piperita* possesses an antiemetic effect, which is beneficial for breast cancer chemotherapy that is frequently accompanied with vomiting [122]. A *Mentha arvensis* EO NE induced early apoptosis in the anaplastic/aggressive thyroid cancer cell line (HTh-7) and exhibited antibacterial activity against *S. aureus*, causing structural changes in the lipid cell membrane of pathogen, with subsequent leakage of cytoplasmic contents [123]. NEs of *Mentha spicata* oil and virgin *Cocos nucifera* oil, in which the ratio of applied oils ranged from 40:60 to 80:20 and which were prepared using Cremophor RH 40 surfactant, showed a strong cytotoxic effect against the oral carcinoma (KON) cell line and have potential to be used as carriers for oral cancer therapy [124]. Lavandin EO obtained from *Lavandula angustifolia* x *L. latifolia* plants containing linalool, eucalyptol, α-pinene, camphor, and linalyl acetate as major components, as well as its NE, exhibited pronounced cytotoxic effects on human neuroblastoma cells (SH-SY5Y) and human lymphoblastic leukemia cells (CCRF-CEM), while human colorectal adenocarcinoma cells (Caco-2), human breast adenocarcinoma cells (MCF-7), and normal breast epithelial cell (MCF1) were more resistant to the treatment; the application of the nanoscale formulation was more effective compared to the free oil, mainly for the treatment of Caco-2 cells [125]. A pectin NE of *Zataria* EO triggered the apoptosis of drug-resistant MDA-MB-231 breast cancer cells and spheroids via rising ROS, loss of mitochondrial membrane potential, and DNA damage, as well as by G_2_ and S-phase arrest, and has potential to be used as an antiproliferative and therapeutic agent in breast cancer therapy [126]. Salehi et al. [127] also reported that an apple pectin-based *Zataria multiflora* essential oil (ZEO) NE considerably suppressed the viability of MDA-MB-231, T47D, and MCF-7 breast cancer cells and greatly induced apoptotic morphological alterations and DNA fragmentation, as well as apoptosis in MDA-MB-231 cells, via loss of mitochondrial membrane potential due to increased ROS accumulation. Moreover, this NE caused G_2_/M cell cycle arrest, DNA strand breakage, and DNA oxidation and interacted with genomic DNA in a minor groove/partial intercalation-binding mode. This NE was recommended for metastatic breast cancer therapy. A NE of *Saccocalyx satureioides* Coss. et Durieu EO having carvacrol, thymol, and γ-terpinene as major constituents showed higher cytotoxicity on liver cancer cells (HepG2) compared to the free EO (106 μg/mL vs 274.8 μg/mL), but lower antioxidant activity, which can be associated with differences in total flavonoid and phenolic content and volatiles in the NE and the free EO. Major constituents of the free EO were borneol, α-terpineol, and thymol [128]. The size of a *Teucrium polium* L. EO NE (12.90 ± 0.04 nm) after loading with oxaliplatin (OXA) increased to 14.47 ± 0.53 nm, and the OXA-loaded NE exhibited synergetic effects in HCT 116 wild-type and HT-29 mutant p53 colon cancer cells, achieving the combination index of 0.94 and 0.88, respectively, and caused a higher percentage of cell apoptosis via mechanism involving ROS-mediated mitochondrial apoptosis compared to the application of monotherapy [129]. An o/w NE prepared using *Tectona grandis* leaf extract with particle size approximately 20 nm showed photodynamic effects, reflected in increased toxicity against melanoma B16 F10 cells under illumination with red light, and showed considerably lower toxicity against normal cells in the dark compared to the free plant extract [130].

A NE of *Jasminum humile* EO containing 24 compounds and *Jasminum grandiflorum* EO containing 17 compounds exhibited anticancer activity and showed lower IC_50_ against HepG2 (26.65 and 22.58 vs 33.96 μg/mL, respectively) and MCF-7 cancer cells (36.09 and 36.19 vs 52.73 μg/mL, respectively) than DOX and was not toxic to normal Vero cells [131]. An optimized NE of *Santolina chamaecyparissus* EO having *trans*-*p*-mentha-2,8-dienol (54.00%), β-cymene (10.16%), *trans*-pinocarveol (6.22%), α-phellandrene (3.74%), pinocarvone (2.86%), borneol (2.09%), and *cis*-jasmone (2.02%) as major components, which showed minimum globule size of 15.98 nm, exhibited stronger anticancer activity against MCF-7 and HepG2 cancer cells and a comparable effect on Caco-2 cells than gemcitabine [132]. The droplet size of an optimized *Pulicaria crispa* EO NE increased after loading with gemcitabine from 9.93 ± 0.53 nm to 11.36 ± 0.0.21 nm, and the gemcitabine-loaded NE showed hundred times higher anticancer activity against MCF-7 and HepG2 cancer cells than the bare drug; synergistic effect observed using a ratio NE:gemcitabine of 1:1 was reflected in 4.48-fold and 2.95-fold increases in apoptosis in MCF-7 and HepG2 cells, respectively, compared to gemcitabine. The drug-loaded NE increased the activation of the intrinsic apoptosis pathway via the upregulation of the expressions of p53 and caspase-3 and the downregulation of BCL-2 expression in MCF-7 cells, whereas the expressions of caspase-3, BAX, and p53 were upregulated in HepG2 cells. The loading of gemcitabine into the *P. crispa* EO NE can reduce the drug dose and eliminate side effects of chemotherapy [133].

A NE of *Linum usitatissimum* seed EO showed considerable cytotoxic effect against human ovarian cancer cells without impacting normal human foreskin fibroblasts (HFF) and caused apoptosis. In addition, the NE showed anti-angiogenic activity reflected in the reduced length and number of blood vessels observed in CAM assay [134]. An *Artemisia vulgaris* EO NE exhibited remarkable cell-selective cytotoxic, apoptotic, and antioxidant activities against MCF-7 cancer cells via upregulating caspase-9, CAT, and SOD gene expression, restrained angiogenesis in MCF-7 breast cancer cells via down-regulating VEGF gene expression, and reduced the number and length of chick CAM blood vessels, suggesting its anti-angiogenic activity [135]. NEs with encapsulated n-hexane or methanol extracts of *Artemisia cina* plant with particle sizes 15–16 nm and neutral surface charge exhibited excellent antiproliferative activity against A549 cells (IC_50_: 12.59 ± 0.7 and 5.6 ± 0.4 μg/mL) compared to free plant extracts (IC_50_: 35.96 ± 1.7 and 41.6 ± 2.8 μg/mL) [136].

A NE encapsulating EO of the medicinal plant *Myrtus communis*, containing α-pinene, eucalyptol, linalyl, linalool acetate, and geranyl acetate as major constituents, with mean droplet size 179 ± 7 nm, which was gelified using carboxymethyl cellulose, exhibited anticancer activity against A-375 melanoma cells with IC_50_: 132.6 μg/mL, whereby the nanogel was 4-folds more effective compared to the bulk EO; the nanogel also exhibited pronouncedly better antimicrobial and antioxidant activity than the bulk EO [137]. A *Syzygium aromaticum* L. EO NE with mean particle size 131.2 nm induced apoptosis of human HT-29 colon cancer cells, and in an in vivo experiment, it exhibited cytoprotective properties on the mice liver, increasing the gene expression of antioxidant enzymes and reducing lipid peroxidation; this NE has potential to be used in colon cancer treatment [138]. A NE of *Syzygium aromaticum* bud EO caused apoptosis and reduced the proliferation of thyroid cancer cells (HTh-7) and exhibited antibacterial activity against *Staphylococcus aureus*, resulting in the leakage of cytoplasmic contents through the destroyed bacterial cell membrane [139]. A NE of frankincense (an aromatic resin obtained from trees of the genus *Boswellia* containing α-pinene as a major volatile compound) with average particle diameter < 20.0 nm prepared with propylene glycol (PG) as a co-surfactant exhibited improved cytotoxic activity against lung cancer A549 cells compared to a PG-free NE, α-pinene, and DOX, being more effective in inducing apoptosis than other formulations and the free EO. The PG-containing frankincense EO NE upregulated the pro-apoptotic genes (DR5, FAAD, caspase-8, p53, and BAX) and downregulated the anti-apoptotic and reoccurrence genes (BCL-2, NF-kB, and STAT-3), whereby it was less cytotoxic to normal WI-38 lung cells [140]. *Zingiber ottensii* EO having zerumbone (25.21%), sabinene (23.35%), and terpinen-4-ol (15.97%) as major constituents was found to be cytotoxic to A549, MCF-7, HeLa, and K562 cells with IC_50_ of 43.37 ± 6.69, 9.77 ± 1.61, 23.25 ± 7.73, and 60.49 ± 9.41 μg/mL, respectively, and induced apoptosis at exposure to 2, 3, and 10 μg EO/mL. The anticancer activity against MCF-7 cells increased after loading the EO in nanoscale formulations, such as NE, ME, nanoemulgel, and microemulgel, and achieved IC_50_ values (expressed in ng of the EO) of 1.08 ± 2.58, 0.74 ± 0.45, 4.31 ± 0.91, and 6.45 ± 5.84 ng/mL, respectively, suggesting a remarkable increase in the efficiency to deliver the EO into MCF-7 cells [141]. Stable NEs fabricated using *Amomum kravanh* EO and olive oil as a fixed oil (an Ostwald ripening inhibitor) applied at a ratio of 80:20 showed a remarkable cytotoxic impact on oral cancer cells, achieving 99.68 ± 0.56% inhibition, and were able to suppress metastasis, causing death of oral cancer cells via the intrinsic apoptosis pathway [142].

A NE of *Nigella sativa* L. EO with particle sizes 20–50 nm pronouncedly reduced the viability and induced apoptosis of MCF-7 breast cancer cells. The treated cancer cells were characterized with membrane blebbing, cytoplasmic vacuolation, marginalization of chromatin, and fragmentation of the nucleus [143]. The pure EO of *Nigella sativa* seeds containing *p*-cymene (40.0%), thymoquinone (31.2%), and trans-α-thujene (12.8%) as major constituents and its NE formulations showing particle sizes ranging from 9.4 to 119.7 nm exhibited dose-dependent antiproliferative activity against hepatocellular carcinoma (HCC) cells HepG2 and Huh-7. An optimized NE fabricated using a single surfactant Tween 80 was the most effective, achieving 78.1% and 90.8% inhibition of HepG2 and Huh-7 cells, respectively, and the estimated respective IC_50_ values of 55.7 and 35.5 μg/mL were lower compared to 100 μg/mL observed with DOX. The apoptotic activity of this NE was higher compared to the pure EO, and the NE also showed a greater upregulation of pro-apoptotic BAX and down-regulation of anti-apoptotic BCL-2 markers with the highest BAX/BCL-2 ratio of 69 against Huh-7 cells, while practically no cytotoxicity against normal WI-38 cells was shown by it, suggesting its potential to be used as an adjuvant liver anticancer agent [144]. An optimized NE of 5^th^ day sprout extract of *N. sativa* L. with average particle size 37.47 nm, which released 98.2% of cargo in 24 h, reduced the viability of hepatocellular carcinoma cells and enhanced the formation and intensity of ROS production and chromatin condensation [145]. A NE fabricated using an *N. sativa* tincture pronouncedly diminished the bioavailability of A2780 ovarian cancer cells, whereby the estimated IC_50_ of 0.72 μg/mL was 34.7-folds lower than that observed against normal umbilical vein endothelial cells (HUVEC) (IC_50_ > 25 μg/mL); the pro-apoptotic effect of the NE was confirmed by acridine orange and propidium iodide staining [146].

A *Pistacia atlantica* fruit EO NE with mean particle size 35.8 nm and zeta potential −32 mV, containing 9% Tween 80 exhibited cytotoxic effects against skin, lung, and prostate cancer (IC_50_ < 10 µg/mL) without adverse impact on normal cells; it enhanced intracellular ROS, caused an increase in the expression of caspase-3, ad caspase-8, and IL-10 genes, and inhibited vessel length and number, as well as cell migration. This EO NE can be considered as an effective therapeutic agent against lung cancer [147]. NEs prepared from *Pinus morrisonicola* needle EO with mean particle size 41.16 nm exhibited a higher inhibition of HT-29 cancer cells compared to normal HFF cells, pronouncedly upregulated caspase-3, caspase-9, VEGF/VEGF-R, CAT, and SOD genes, and caused the apoptotic death of cancer cells, which was reflected in increased sub-G1 peaks [148]. A NE of *Citrus aurantium* bloom EO containing linalyl acetate, limonene, and α-terpineol as major constituents with average particle size 76.9 ± 6.11 nm and zeta potential −43.5 mV exhibited cytotoxic impact on A549 cells (IC_50_: 152 μg/mL), induced the overexpression of caspase-3, and triggered apoptosis. Moreover, the NE practically did not show remarkable histopathological alteration in the liver and kidney but enhanced the jejunum morpho-structural architecture and hepatic antioxidant redox potential in mice receiving daily NE doses of 10 and 20 mg/kg body weight via gavage for 30 days. It was assumed that this NE could be used as an alternative to prevent lung cancer progression [149]. A NE of *Drimys brasiliensis* EO containing bicyclogermacrene (19.6%) and cyclocolorenone (18.2%) as major constituents with particle size 168 nm and zeta potential ca. –34 mV reduced the viability of human glioblastoma U-138 MG and human bladder carcinoma T24 cells and caused the late apoptosis of the cancer cells [150]. A NE of *Ricinus communis* EO showing droplets of 81.4 nm and exhibiting superb antioxidant activity considerably reduced the viability of HepG2 cells after incubation for 48 h, and with increasing NE doses, an upregulation of the expression of caspase-3 and an increase in sub-G1 peaks in treated cancer cells were observed. The impact of the NE was cell-specific; normal L929 cells were not affected [151].

## 4. Essential Oils Encapsulated in Liposomes

**Figure 5:**
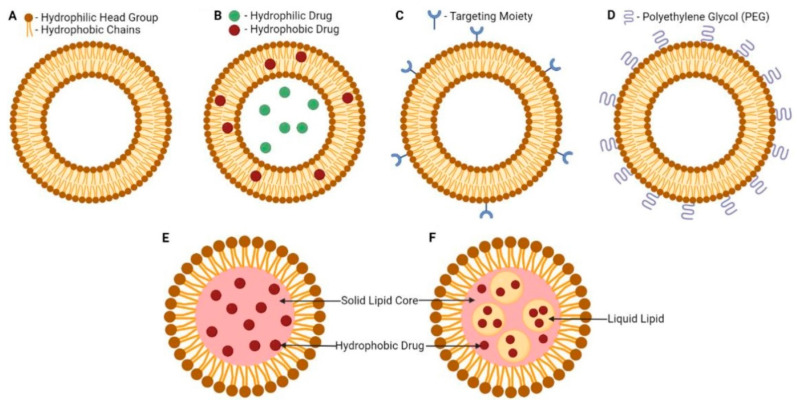
Schematic illustrations of lipid-based nanosystems: liposome (**A**), drug-loaded liposome (**B**), targeted liposome (**C**), PEGylated liposome (**D**), solid lipid nanoparticle (**E**), and nanostructured lipid carrier (**F**). Adapted from [67].

Liposomes are small artificial spherical vesicles formed mostly by a lipid bilayer and an inner compartment isolated from the environment. Taking into account their size, biocompatibility, and hydrophobic and hydrophilic properties, liposomes are ideal as drug delivery systems. They are mostly prepared from natural or synthetic phospholipids, often with the addition of cholesterol to strengthen the membrane, most commonly by extrusion, injection, or microfluidic methods. From the chemical point of view, liposomes are formed by phospholipids enriched with phosphatidylcholine. On their external surface, there can also be `ligands required for the recognition and acceptance of the liposome by a particular tissue. Main types of liposomes include multilamellar vesicles, small unilamellar vesicles, and large unilamellar vesicles. The number of concentric membranes in the liposome depends on the conditions of its formation. However, bilayer or single-layer membranes are most common [152,153,154]. Schematic illustrations of lipid-based nanosystems including liposomes, SLNPs, and nanostructured lipid carriers (NLCs) are shown in Figure 5 [67].

An improvement of the bioavailability of phenolic compounds applied as antidiabetic, anti-inflammatory, and anticancer agents by their encapsulation into liposomes was overviewed by Tatipamula and Kukavica [155]. Progress in anticancer phytochemical-loaded liposomal formulations with improved therapeutic effectiveness due to enhanced entry across cell barriers and cancer-specific targeting capabilities was comprehensively reviewed by Chavda et al. [156]. Benefits of plant-derived compounds encapsulated in liposomes and nanoliposomes ensuring their improved stability and bioavailability while used in the pharmaceutical and nutraceutical industry were summarized by Jahadi et al. [157].

### 4.1. Isolated Components of Essential Oils

Liposomes of furanodiene, a primary sesquiterpene extracted from the rhizome EO of *Curcuma wenyujin*, inhibited the in vitro proliferation of twelve tested cancer cell lines, including HeLa, Hep-2, HL-60, and U251 cells, as well as the proliferation of uterine cervix (U14) tumor induced in mice in vivo, where tumor inhibition rates achieved even 58.29% after intraperitoneal administration of a dose 80 mg/kg [158]. Long-circulating liposomes co-encapsulating β-elemene (a volatile compound of *Rhizoma curcumae* EO) and IR780 photosensitizer with mean particle size 130 nm and high encapsulation efficiency (EE) for both encapsulated compounds showed a superb photothermal conversion efficiency upon near infrared (NIR) light irradiation and, after i.v. administration, gradually accumulated in the tumor area, causing an increase in tumor temperature by 20 °C under irradiation with an 808 nm laser. At exposure to NIR light, the liposomal formulation with co-encapsulated β-elemene and IR780 generated remarkable ROS amounts and exhibited improved cytotoxicity against Lewis lung cancer cells compared to the non-irradiated nanoformulation or laser-irradiated IR780-encapsulating liposomes [159].

### 4.2. Essential Oils and Plant Extracts

A liposomal formulation of bergamot EO increased the anticancer activity of the free EO in vitro against human SH-SY5Y neuroblastoma cells [160]. The comparison of nanosized liposomes and nanoniosomes with a negative charge that encapsulated *Achillea millefolium* EOs showed that the nanoliposomes were able to encapsulate a higher percentage of EOs than the nanoniosomes, although the nanoniosomes demonstrated a smaller size and slower release than the nanoliposomes. The nanoformulations showed excellent antimicrobial effect exceeding that of the free EO and have potential to be used in the treatment of breast cancer [161]. Based on the investigation of the viability of MCF-7 cancer cells, the 24-h IC_50_ of 25 μg/mL was estimated for free *Origanum vulgare* L. EO, while by encapsulation of the EO into Phospholipon^®^ 90H liposomes, the cytotoxic activity was considerably enhanced and the reduction of cell viability to 25.89% was observed compared to 50.10% reduction determined for the free EO. On the other hand, the reduction of cell viability observed with the application of *O. vulgare* EO-loaded Phospholipon^®^ 85G or *O. vulgare* EO-loaded Lipoid S100 liposomes (51.22% and 40.41%, respectively) was comparable with that of the free EO [162]. The improved anticancer properties of a nanoliposomal system containing *Rosmarinus officinalis* EO against MCF-7 cells compared to the free EO described by Salari and Salari [163] were achieved due to ameliorated drug delivery. A nanoliposomal formulation of the aqueous extract of *Agrostemma githago* seeds with mean particle size 171.5 nm showed considerably higher cytotoxicity against the AGS human gastric cancer cell line than the free extract (IC_50_: 4.43 ± 1.49 μg/mL vs 13.02 ± 0.95 μg/mL). Considering that agrostin and saponin are the most important compounds in the extract showing cytotoxic effect, it can be supposed that the ability of saponin to increase the entry of agrostin into target cancer cells was escalated due to encapsulation into liposomes [164].

Nanoliposomes prepared using dipalmitoylphosphatidylcholine (DPPC), polyethylene glycol (PEG) 2000, 1,2-distearoyl-sn-glycero-3-phosphorylethanolamine (DSPE), and cholesterol, which encapsulated raw extract of *Bistorta amplexicaulis* and had particle sizes 140–155 nm, zeta potential from −16.9 to −19.8 mV, and 81% EE, exhibited pronouncedly higher uptake and cytotoxicity against MCF-7 breast cancer cells and HepG2 hepatocellular carcinoma cells in vitro compared to the free extract and reduced toxicity against human umbilical vein endothelial cells (HUVEC) [165]. *Brucea javanica* oil-loaded liposomes inhibited the proliferation of HepG2 cells in a dose-dependent manner via inducing apoptosis [166]. The intravenous administration of progesterone-like compounds from the leaf extract of *Dendrophthoe pentandra* L. encapsulated in 10% liposomal small unilamellar vesicles using doses 3, 5, and 7 mg liposomal formulation per 100 g body weight of rats resulted in 1.20–2.40 fold higher plasma concentration of the active compounds compared to the control [167].

## 5. Essential Oils Encapsulated in Solid Lipid Nanoparticles

Solid lipid nanoparticles (SLNPs) are similar to oil in water emulsions, but at room temperature, the liquid emulsion part is replaced by a solid lipid part, which allows loading both hydrophilic and hydrophobic molecules [168,169,170]. Elkordy et al. [83] comprehensively overviewed pharmaceutical formulations prepared from therapeutically active extracts of natural products. Phytonanoformulations exhibiting the controlled release of active constituents and ensuring their enhanced absorption at pancreatic cancer sites resulting in improved therapeutic effects on cancer cells were discussed by Gupta et al. [171]. Nanoformulations containing vegetable oil-based bioactive compounds showing nutraceutical and human health-supporting properties showed ameliorated uptake, absorption, and bioavailability of these compounds in the body and can contribute to the prevention and management of diseases [172]. In addition, the drug delivery of natural products, including extracts/EOs of nine medicinal plants and nine natural bioactive compounds using nanocarriers, which were designed for powerful breast cancer treatment, was overviewed by Yap et al. [173].

### 5.1. Isolated Components of Essential Oils

Cuminaldehyde-loaded gelled SLNPs prepared using a hot emulsification process with monoglyceride as a lipid gelator and showing particle sizes 117–138 nm exhibited a stronger cytotoxic impact on human lung and colorectal cancer cells than free cuminaldehyde, and the formulation showed minor toxicity against normal peripheral blood mononuclear cells [174]. Linalool-loaded SLNPs consisting of myristyl myristate, cetyl esters, and cetyl palmitate, which were fabricated by sonication in the presence of Pluronic^®^, with particle sizes 90–130 nm, zeta potential approx. −4.0 mV and >80% EE exhibited the controlled release of linalool for 72 h and more effectively inhibited proliferation of hepatocarcinoma HepG2 and lung adenocarcinoma A549 cells than free linalool [175].

### 5.2. Essential Oils and Plant Extracts

Sharifalhoseini et al. [176] prepared SLNPs encapsulating *Foeniculum vulgare* EO with mean particle size 55.43 nm and zeta potential −29.54 ± 11.67 mV, which exhibited strong toxicity against MCF-7 cells and induced apoptosis in cancer cells, while their toxicity against normal HUVECs cells was low. SLNPs loaded with *Ferula assa foetida* seed oil pronouncedly suppressed the growth of human NTERA-2 embryocarcinoma cells; induced apoptotic death via upregulating the expression of TNF-α, P21, and caspase-3 genes; and inhibited angiogenesis in chorioallantoic membrane (CAM) tissue via reducing the length and number of its blood vessels [177].

While with the application of 1200 μg/mL of *Mentha longifolia* and *Metha pulegium* EOs, the viability of a melanoma cell line (A-375) and breast cancer cells MDA-MB-468 and MCF-7 achieved > 55%, after the encapsulation of the EOs into SLNPs with mean particle sizes 107 ± 9 and 191 ± 8 nm and zeta potentials −7.10 and −4.81 mV, respectively, a dose of 600 μg/mL reduced the viability of the tested cell lines approximately to 10% [178]. SLNPs encapsulating *Zataria multiflora* EO with mean particle size 176 ± 8 nm and 67 ± 5% EE showed antiproliferative effect on MDA-MB-468 and A-375 cancer cells in a dose-dependent manner, where a dose of 75 μg/mL reduced their viabilities to <13% [179]. *Satureja khuzistanica* EO-loaded SLNPs, the surface of which was modified with folate-bound chitosan, exhibited selective toxicity against MCF-7 cells with IC_50_ 88 μg/mL, and it was found that these SLNPs inhibited cancer cells via activating the internal pathway of apoptosis, as well as cell cycle disruption [180].

SLNPs encapsulating *Pistacia atlantica* EO with particle sizes ranging from 92.20 ± 2.1 nm to 334.5 ± 3.2 nm, negative zeta potential values, 97.3% EE, and 9.6% loading capacity not only inhibited the proliferation of MDA-MB-231 cells, but also stimulated apoptosis in these breast cancer cells, and, in contrast to control or placebo groups, considerably reduced the number of cells in the G_2_/M phase [181].

## 6. Essential Oils Encapsulated in Nanostructured Lipid Carriers

Nanostructured lipid carriers (NLCs) are systems for drug administration consisting of solid and liquid biocompatible and biodegradable lipids forming a basic matrix, surface active substances, and co-surfactants [182,183,184,185]. NLCs can be understood as a next generation of SLNP carriers. Their properties and production are described in detail by Chauhan, Elmowafy, and Fang [184,185,186]. Innovative NLC drug delivery systems suitable to be loaded with natural plant extracts and their possible biomedical applications were discussed by Rahman, et al. [187].

### 6.1. Isolated Components of Essential Oils

Eucalyptol encapsulating NLCs fabricated using the high pressure homogenization technique with average particle size 71.8 ± 2.1 nm and zeta potential −2.927 ± 0.163 mV exhibited cytotoxic effects on human (MDA-MB-231) and murine (4 T1) breast cancer cells in vitro (72-h IC_50_ values of 10.00 ± 4.81 μg/mL and 17.70 ± 0.57 μg/mL, respectively) and induced apoptosis in the MDA-MB-231 cells. In an in vivo sub-chronic toxicity study using a BALB/c mice model, the eucalyptol-containing nanoformulation did not cause toxicity or mortality to animals, and changes observed in the mice body weight, hepatic, and renal histopathology, as well as NO and malondialdehyde contents were negligible [188].

### 6.2. Essential Oils

NLCs encapsulating either lavender or melaleuca EO and bupivacaine (S(-)75:R(+)25) showed cytotoxic effects on mice (B16-F10) and human (SK-MEL-25) melanoma cells and reduced the relative IC_50_ values by 80% and 62% at application of the lavender EO and by 80% and 25% using the melaleuca EO compared to free bupivacaine; moreover, the anesthesia time of encapsulated bupivacaine was doubled [189]. NLCs loaded with *Pistacia atlantica* Desf EO showing spherical shape with size 151 nm and negative zeta potential –29.1 ± 1.4 mV reduced the viability of SK-BR-3 breast cancer cells via cell cycle arrest and apoptosis and may be used for breast cancer therapy [190].

## 7. Essential Oils and Their Components with Anticancer Activity

Within this contribution, EOs and their components incorporated into various lipid-based delivery nanosystems are described. Table 1 summarizes the discussed EOs and individual secondary metabolites isolated from EOs or herbal extracts that have been observed to have in vitro anticancer activity when incorporated into nanosystems.

## 8. Conclusions

Natural compounds are once again becoming an important source of inspiration for scientists to design new anti-invasive drugs. The natural compounds themselves often have a complex structure or disadvantageous properties (bioavailability, stability), so they are predominantly considered as lead compounds only. However, small molecules such as essential oils found in many plants are not only a frequent source of inspiration, but also a commonly used therapeutic agent. Traditionally, various EOs are used as antimicrobial compounds or antioxidants, but due to their disadvantageous properties, such as volatility, irritation, and limited bioavailability, they are rarely used as real drugs and only as supplements in the treatment. However, these compounds have also been found to have therapeutic efficacy for difficult-to-treat diseases, such as cancer. They can reduce the unwanted side effects of treatment, and their use with other chemotherapeutics can prevent the selection of cancer cells resistant to treatment. A secondary benefit associated with the use of natural compounds is the possibility to reduce the use of substances harmful to health and the environment, such as toxic reagents or harmful solvents. The application of innovative technologies and advanced drug forms, i.e., the incorporation of bioactive agents of natural origin into nanoformulations, preferably with targeted biodistribution, makes it possible to overcome some of the physicochemical limitations of these drugs and enables their evaluation in terms of their anticancer activity in vitro and in vivo, thus far mainly in animal models. As already mentioned, many nanoformulations of EOs have demonstrated in vitro potential as anticancer agents, but there is still a long way to go before successful registration and application of these traditional compounds from folk medicine as anticancer drugs.

## Figures and Tables

**Table 1:** In vitro anticancer effects of nanoformulated EOs and individual secondary metabolites isolated from EOs or herbal extracts.

Formulation	Plant EO or Constituent of EO	Tested Human Cancer Cell Lines	Refs.
NEs	carvacrol	lung adenocarcinoma A549 cells	[104]
	carvacrol	doxorubicin resistant-A549 cells	[106]
	*Carum carvi* EO	HT-29 colorectal adenocarcinoma cells	[111]
	*Cuminum cyminum* seed EO	SAS tongue carcinoma cells	[112]
	*Anethum graveolens* EO	lung adenocarcinoma A549 cells	[114]
	*Ferula assa-foetida* EO	MCF-7 breast cancer cells, mammary cancer tissue	[116]
	*Ferula gummosa* EO	HT-29 colorectal adenocarcinoma cells	[117]
	*Heracleum persicum* EO	MDA-MB-231 breast cancer cells	[118]
	*Apium graveolens* EO	SAS tongue carcinoma cells	[119]
	*Origanum vulgare* EO	PC3 prostate cancer cells	[120]
	*Origanum glandulosum* Desf. EO	HepG2 liver cancer cells	[121]
	*Mentha piperita* EO	MCF-7, MDA-MB-231, MDA-MB-468 breast cancer cells	[122]
	*Mentha arvensis* EO	HTh-7 thyroid cancer cells	[123]
	*Mentha spicata* EO	KON oral squamous carcinoma cells	[124]
	Lavandin EO	MCF-7 breast cancer cells, CCRF-CEM lymphoblastic leukemia cells, Caco-2 colorectal adenocarcinoma cells	[125]
	*Zataria* EO	MCF-7, MDA-MB-231 breast cancer cells	[126]
	*Zataria multiflora* EO	MCF-7, MDA-MB-231 and T47D breast cancer cells	[127]
	*Saccocalyx satureioides* Coss. et Durieu EO	HepG2 liver cancer cells	[128]
	*Teucrium polium* L. EO	HCT 116 and HT-29 colorectal adenocarcinoma cells	[129]
	*Jasminum humile* EO	HepG2 liver cancer cells, MCF-7 breast cancer cells	[131]
	*Jasminum grandiflorum* EO	HepG2 liver cancer cells, MCF-7 breast cancer cells	[131]
	*Santolina chamaecyparissus* EO	MCF-7 breast cancer cells, HepG2 liver cancer cells, Caco-2 colorectal adenocarcinoma cells	[132]
	*Pulicaria crispa* EO	MCF-7 breast cancer cells, HepG2 liver cancer cells	[133]
	*Linum usitatissimum* seed EO	A2780 ovarian cancer cells	[134]
	*Artemisia vulgaris* EO	MCF-7 breast cancer cells	[135]
	*Artemisia cina* EO	lung adenocarcinoma A549 cells	[136]
	*Myrtus communis* EO	A-375 melanoma cells	[137]
	*Syzygium aromaticum* EO	HT-29 colorectal adenocarcinoma cells	[138]
	*Syzygium aromaticum* buds EO	HTh-7 thyroid cancer cells	[139]
	frankincense resin	lung adenocarcinoma A549 cells	[140]
	*Zingiber ottensi* EO	MCF-7 breast cancer cells	[141]
	*Nigella sativa* EO	MCF-7 breast cancer cells.	[143]
	*Nigella sativa* EO	HepG2 and Huh-7 liver cancer cells	[144]
	*Pistacia atlantica* EO	lung adenocarcinoma A549 cells	[147]
	*Pinus morrisonicola* needle EO	HT-29 colorectal adenocarcinoma cells	[148]
	*Citrus aurantium* bloom EO	lung adenocarcinoma A549 cells	[149]
	*Drimys angustifolia* EO	U-138 MG glioblastoma cells, T24 bladder carcinoma cells	[150]
	*Ricinus communis* EO	HepG2 liver cancer cells	[151]
Liposomes	*Curcuma wenyujin* EO	HeLa cervical cancer cells, laryngocarcinoma Hep-2 cells, HL-60 promyelocytic leukemia cells, U251 human glioma cells	[158]
	*Curcuma longa* EO	Lewis lung cancer cells	[159]
	*Citrus bergamia* EO	SH-SY5Y neuroblastoma cells	[160]
	*Achillea millefolium* EOs	MCF-7 breast cancer cells	[161]
	*Origanum vulgare* L. EO	MCF-7 breast cancer cells	[162]
	*Rosmarinus officinalis* EO	MCF-7 breast cancer cells	[163]
	*Brucea javanica* EO	HepG2 liver cancer cells	[166]
SLNPs	cuminaldehyde	lung adenocarcinoma A549 cells, HCT 116 colorectal adenocarcinoma cells	[174]
	linalool	HepG2 liver cancer cells, lung adenocarcinoma A549 cells	[175]
	*Foeniculum vulgare* EO	MCF-7 breast cancer cells	[176]
	*Ferula assa-foetida* seed EO	NTERA-2 embryocarcinoma cells	[177]
	*Mentha longifolia* EO	MDA-MB-468 and MCF-7 breast cancer cells	[178]
	*Mentha pulegium* EO	MDA-MB-468 and MCF-7 breast cancer cells	[178]
	*Zataria multiflora* EO	MDA-MB-468 breast cancer cells, A-375 melanoma cells	[179]
	*Satureja khuzistanica* EO	MCF-7 breast cancer cells	[180]
	*Pistacia atlantica* EO	MDA-MB-231 breast cancer cells	[181]
NLCs	eucalyptol	MDA MB-231 breast cancer cells	[188]
	lavender EOs	SK-MEL-25 melanoma cells	[189]
	*Pistacia atlantica* Desf EO	SK-BR-3 breast cancer cells	[190]

## Data Availability

Not applicable.
